# Melatonin protects skin keratinocyte from hydrogen peroxide-mediated cell death *via* the SIRT1 pathway

**DOI:** 10.18632/oncotarget.7679

**Published:** 2016-02-24

**Authors:** Ju-Hee Lee, Ji-Hong Moon, Uddin MD. Nazim, You-Jin Lee, Jae-Won Seol, Seong-Kug Eo, John-Hwa Lee, Sang-Youel Park

**Affiliations:** ^1^ Biosafety Research Institute, College of Veterinary Medicine, Chonbuk National University, Jeonju, Korea

**Keywords:** melatonin, autophagy, hydrogen peroxide, keratinocyte, Pathology Section

## Abstract

Melatonin (N-acetyl-5-methoxytryptamine), which is primarily synthesized in and secreted from the pineal gland, plays a pivotal role in cell proliferation as well as in the regulation of cell metastasis and cell survival in a diverse range of cells. The aim of this study is to investigate protection effect of melatonin on H_2_O_2_-induced cell damage and the mechanisms of melatonin in human keratinocytes. Hydrogen peroxide dose-dependently induced cell damages in human keratinocytes and co-treatment of melatonin protected the keratinocytes against H_2_O_2_-induced cell damage. Melatonin treatment activated the autophagy flux signals, which were identified by the decreased levels of p62 protein. Inhibition of autophagy flux via an autophagy inhibitor and ATG5 siRNA technique blocked the protective effects of melatonin against H_2_O_2_-induced cell death in human keratinocytes. And we found the inhibition of sirt1 using sirtinol and sirt1 siRNA reversed the protective effects of melatonin and induces the autophagy process in H_2_O_2_-treated cells. This is the first report demonstrating that autophagy flux activated by melatonin protects human keratinocytes through sirt1 pathway against hydrogen peroxide-induced damages. And this study also suggest that melatonin could potentially be utilized as a therapeutic agent in skin disease.

## INTRODUCTION

H_2_O_2_isobserved in nearly all types of oxidative stress and oxygen radicals, spreads freely within and beyond cells and tissues [1] and has the ability to modulate various signal transduction pathways [2, 3]. When in an environment with keratinocytes, H_2_O_2_-induced cell damage [4] can include early apoptosis and oxidative stress. Beyond this, H_2_O_2_, which is generated in the skin as a response to UV radiation, is capable of inducing lipid peroxidation, protein modification and DNA damage [5] as well as producing oxygen radicals that cause direct oxidative stress in human keratinocytes [6]. H_2_O_2_-induced oxidative stress in keratinocytes can trigger many skin diseases, including vitiligo [5], skin cancer and advanced skin aging [7]. Consequently, these processes have drawn considerable attention in the dermatology field.

Melatonin, N-acetyl-5-methoxytryptamine, is a lipophilic molecule that is mainly synthesized in the pineal gland and functions as a ubiquitous physiological mediator [8]. It has been found in a diverse array of tissues, including the intestine, thymus, gastrointestinal tract [9], eyes [10], kidneys [11], lungs [12], skin [13] and immune system [14], among others, with most of these tissues expressing melatonin-synthesizing enzymes [15-17]. Numerous studies have recognized melatonin as an important factor in protective effects, including the regulation of circadian rhythms, the reduction of cancer or other metastatic diseases, and increased anti-inflammatory properties and function of free radical scavenger as well as cell proliferation [18-22] in many different cells [23, 24]. In human keratinocytes, melatonin and its metabolites [25] play a pivotal role in protecting several vital physiological and pathological processes [26] and regulating immune and endocrine functions [27]. It also has many other effects by acting as a hormone, neurotransmitter, cytokine and biological modifier [28]. Additionally, melatonin exhibits protective effects on H_2_O_2_-induced cytotoxicity [29] and has been identified as a regulator of autophagy [30, 31] in mTOR-dependent pathways [32].

Autophagy is recognized as the cellular response involved in maintaining homeostasis and defending against infection [33]. This response also triggers antigen presentation [34],embryogenesis [35], development [36] and metabolism [33] through the degradation of unnecessary and long-lived proteins, cytosolic components, mitochondria [37], macromolecules, and damaged organelles [38]. One such protein is sequestosome-1, p62, a molecular adapter that interfaces between autophagic machinery and its substrates [39, 40]. The degradation process is initiated by generating double-membrane organelles, which then lead to the formation of autophagosomes. Subsequently, the autophagosomes fuse with lysosomes to form autolysosomes, where components are then digested [41, 42]. This process leads to the recycling of components back into the cytoplasm [43]. Autophagy, which is constitutively induced in response to basal level environmental signals, is a very important function because it maintains cellular quality in protein conformational disorders and promotes rapid adaptation to microenvironmental changes [44-46].

Silent information regulator 1 (Sirt1), a NAD-dependent class III histone deacetylase is a type of histone deacetylase [47, 48]. Sirt1 has an influence on keratinocytes, adipocyte, muscle, liver, and endocrine pancreas physiology [49-54]. Sirt1 in those tissues plays a key role in the longevity effects elicited by calorie restriction [55, 56]. And also modulation of sirt1 activity implicated in signaling networks, regulating cell metabolism, cellular senescence, endocrine signaling, stress responses and controlling cell death and survival [51, 52, 57, 58]. In addition, sirt1 has been shown to inhibit cell differentiation in white adipocytes [59] and keratinocytes [53]. As cellular targets for sirt1, there are acetylated p53 [57, 58], p300, Ku70 [60] forkhead (FOXO) transcription factors [61, 62], PPARγ [50] and PPARγ coactivator-1α (PGC-1α) protein [51]. Deacetylation of p53 transcription factors and FOXO inhibits cell damage including apoptosis and enhances the cell survival signal [58, 60, 62]. Also it is known that melatonin can induce the increase of sirt1, which then leads to the deacetylation and subsequent inhibition of p53. However, the role of sirt1 in the protective effect of melatonin against H_2_O_2_-induced keratinocyte death has not been explored.

Previous studies have reported melatonin's role in inducing autophagy [30, 63]; however, no investigation has taken place regarding melatonin's possible role in regulating the sirt1/autophagy process in keratinocytes. The present study demonstrates that melatonin enhances the autophagy process as a primary mechanism through sirt1 activation in protecting keratinocytes against hydrogen peroxide-induced cell damage. These results suggest that melatonin may play a pivotal role as a therapeutic target for skin disease.

## RESULTS

**Figure 1:**
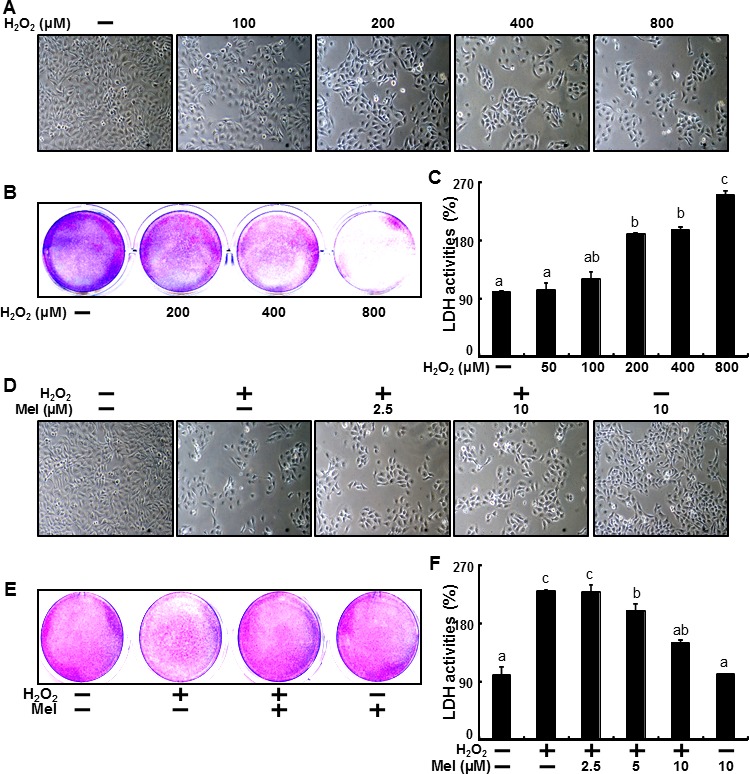
Melatonin prevents hydrogen peroxide-induced human keratinocyte cell death HaCaT keratinocytes were exposed to 100, 200, 400 and 800 μM of H_2_O_2_ for 18 hr. Treated cells were photographed using light microscopy **A.** and cell viability was measured *via* crystal violet staining. Viability of the control cells was set at 100% and viability relative to the control was measured **B.**. LDH activity was measured by quantifying LDH released in the medium **C.**. HaCaT keratinocytes were pretreated with melatonin (2.5, 5, and 10 μM) for 12 hr and exposed to 800 μM of H_2_O_2,_ for 18 h. Treated cells were photographed using light microscopy **D.** and cell viability was measured *via* crystal violet staining. Viability of the control cells was set at 100% and viability relative to the control was measured **E.**. LDH activity was measured by quantifying LDH released in the medium **F.**. The data were analyzed using ANOVA and Tukey multiple range tests (*P* < 0.01). Means sharing a common alphabetical symbol did not significantly differ.

H_2_O_2_'s relationship to cell damage in human keratinocytes is well known [64, 65] and results from the present study confirmed this. HaCaT keratinocytes were exposed to H_2_O_2_ for 18 h, after which we confirmed cell morphology and cell number using a microscope (Figure 1A) and assessed cell viability using crystal violet assay (Figure 1B). Following the H_2_O_2_ treatment, we observed a gradual decrease in the number of cells as well as decreases cell survival rates. Consistent with these results, LDH activity increased in the presence of H_2_O_2_ (Figure 1C).

Recent studies have demonstrated that melatonin can facilitate preventive effects [66, 67] in keratinocytes and reduce cell damage related to the oxidative stress of hydrogen peroxide in human keratinocytes [68], but the effects of melatonin on hydrogen peroxide-induced cell death are unclear. Thus, we examined the preventive effects of melatonin against H_2_O_2_-induced cell death. HaCaT keratinocytes were pre-treated with melatonin at concentrations of 2.5, 5 and 10 μM for 12 hr and then exposed to H_2_O_2_ for 18 hr. After this, we confirmed cell morphology and cell numbers using a microscope (Figure 1D). And Cell viability also showed that melatonin prevented H_2_O_2_-induced cell death (Figure 1E). Moreover, increases in the LDH activity of H_2_O_2_-treated keratinocytes were gradually decreased with melatonin (Figure 1F).

**Figure 2:**
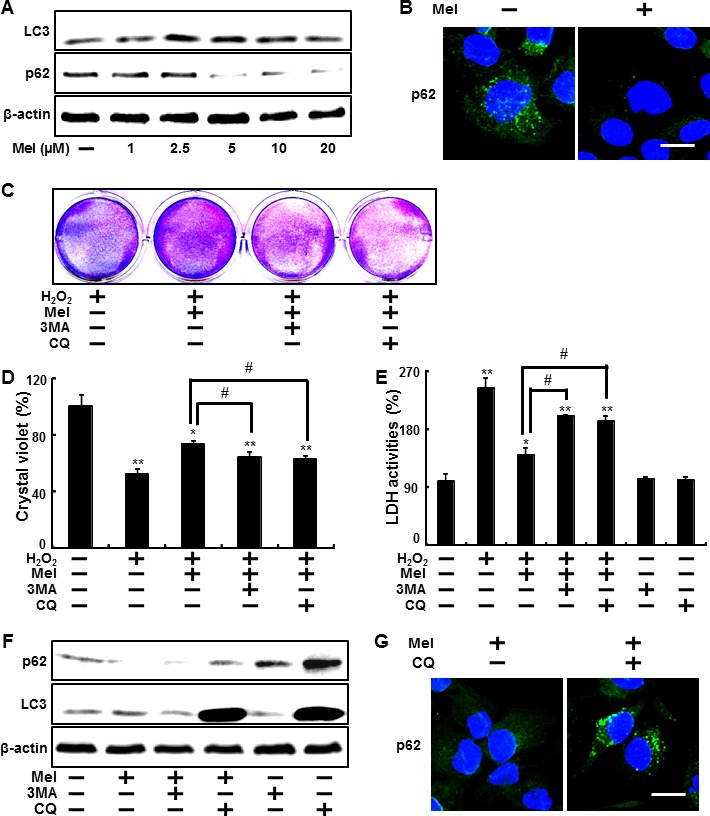
Melatonin-induced autophagy prevents the hydrogen peroxide-mediated human keratinocytes damage HaCaT keratinocytes were treated with melatonin at different concentrations (1∼20 μM) with or without H_2_O_2_ (800 μM) for 24 hr. Western blot analysis of LC3-II and p62 proteins was conducted from the HaCaT keratinocytes **A.**. The melatonin-treated cells were immunostained with DAPI (blue) and p62 antibody (green) and fluorescence was examined **B.**. HaCaT keratinocytes were pretreated with melatonin (10 μM) for 12 hr in the presence of autophagy inhibitors, 3MA (1 mM) and CQ (10 μM), and then exposed to 800 μM of H_2_O_2_ for 18 hr. Cell viability was measured using crystal violet staining. Viability of the control cells was set at 100% and viability relative to the control was measured **C. D.**. LDH activities were measured by quantifying LDH released in the medium **E.**. HaCaT keratinocytes were pretreated with melatonin (10 μM) in the presence of 3MA (1 mM) and CQ (10 μM) and then exposed to 800 μM of H_2_O_2_ for 12 h. Western blot analysis of LC3-II and p62 proteins was conducted from the HaCaT keratinocytes. β-actin was used as the loading control **F.**. HaCaT keratinocytes were analyzed by immunocytochemistry for p62 **G.**. The cells were immunostained with p62 antibody (green) and observed in the fluorescent view. Scale bar: 10 μm. The bar graph indicates the mean ± standard error of the mean (SEM) (*n* = 4). * *p* < 0.05 and ** *p* < 0.01, represent significant differences between the control and each treatment group and # *p* < 0.01; significantly different when compared with H_2_O_2_ and melatonin-treated group.

Many studies have reported that melatonin increases autophagy [69] and that induction of autophagy is responsible for protective mechanisms in various cells. Therefore, we sought to verify whether melatonin influenced H_2_O_2_-induced cell death *via* autophagy. To clarify the mechanisms behind melatonin, we first examined the effects of melatonin treatment on the conversion of LC3-I to LC3-II. HaCaT keratinocytes treated with 1, 2.5, 5, 10 μM of melatonin for 24 hr were subjected to Western blot analysis and the conversion of LC3-I to LC3-II and p62 protein levels were analyzed. We found a gradual increase in LC3-II expression and p62 degradation in melatonin-treated cells. A density graph of protein levels in p62 also reflected dose-dependent changes (Figure 2A). Immunocytochemistry demonstrated decreased p62 immunoreactivity in melatonin-treated keratinocytes as compared to the control (Figure 2B).

To determine the impact of melatonin-induced autophagy on H_2_O_2_-linked cell death in HaCaT keratinocytes, we blocked autophagy using the autophagy inhibitors 3-Methyladenine (3MA) and chloroquine (CQ). HaCaT keratinocytes pre-treated with autophagy inhibitor for 1 h were exposed to melatonin 10 μM in the presence of H_2_O_2_. On crystal violet assay and LDH assay, cells treated with melatonin in the presence of 3MA and CQ displayed significantly decreased cell viability compared to treated melatonin treatment with H_2_O_2_ (Figures 2C and 2D). In Figure 2E, we examined the decreased LDH activities released to media by melatonin against H_2_O_2_ were restored by autophagy inhibitors. Treatment with 3MA and CQ did not cause any adverse effects on cell viability (data not shown). HaCaT keratinocytes treated with 10 μM of melatonin in the presence of 3MA and CQ for 24 hr were subjected to Western blot analysis, after which LC3-I to LC3-II conversion and p62 protein levels were analyzed. According to these protein levels, 3MA blocked melatonin-enhanced conversion of LC3-I to LC3-II and reduced p62 degradation, respectively, and CQ also decreased p62 protein levels (Figure 2F). And also p62 immunoreactivity reduced by melatonin was restored in CQ-pretreated keratinocytes (Figure 2G).

**Figure 3:**
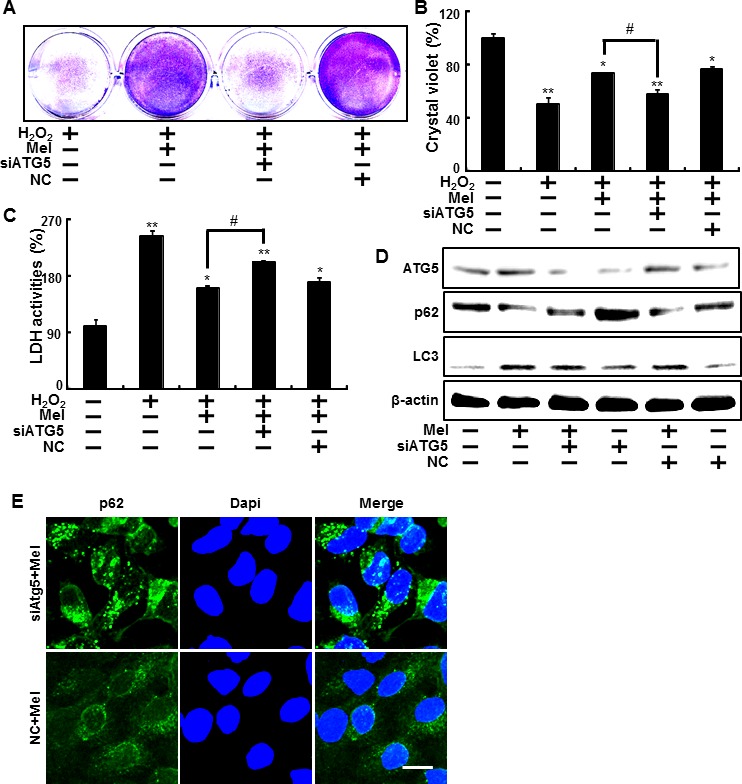
Inhibition of autophagy using ATG5 siRNA to counteract melatonin-related autophagy increases ATG5 small interfering RNA (ATG5 siRNA) or negative control siRNA (NC siRNA) transfected HaCaT keratinocytes were incubated with 800 μM H_2_O_2_ for 18 hr in the presence of melatonin. Cell viability was measured using crystal violet staining. Viability of the control cells was set at 100% and viability relative to the control was measured **A. B.**. LDH activity was measured by quantifying LDH released in the medium **C.**. ATG5 siRNA or NC siRNA transfected HaCaT keratinocytes were incubated with melatonin for 24 hr. Western blot analysis of LC3-II and p62 proteins was conducted from the HaCaT keratinocytes. β-actin was used as a loading control **D.**. HaCaT keratinocytes were analyzed by immunocytochemistry for p62 **E.**. The cells were immunostained with p62 antibody (green) and observed in the fluorescent view. Scale bar: 10 μm. The bar graph indicates the mean ± standard error of the mean (SEM) (*n* = 4). * *p* < 0.05 and ** *p* < 0.01, represent significant differences between the control and each treatment group and # *p* < 0.01; significantly different when compared with H_2_O_2_ and melatonin-treated group.

We then sought to determine whether the inhibition of autophagy using ATG5 siRNA influences melatonin's protective effects against hydrogen peroxide. Results from the crystal violet staining indicate that the knockdown of autophagy by ATG5 siRNA significantly increased cell death (Figure 3A and 3B). In the same manner, the knockdown of ATG5 enhanced LDH activity, which was reduced by melatonin (Figure 3C). LC3-II proteins increased as a result of melatonin were reduced and decreased p62 levels due to melatonin were restored to control conditions, where the cell was transfected to ATG5 siRNA (Figure 3D). In the same manner, ICC showed the silencing ATG5 using ATG5 siRNA restored the melatonin-induced p62 reduction to control condition (Figure 3E). These data indicated that the knockdown of ATG5 expression blocks melatonin's protective effects, which inhibits human keratinocyte damage.

**Figure 4:**
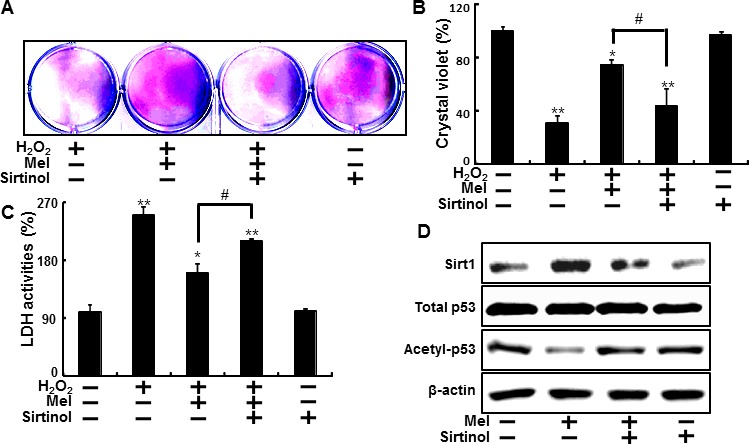
Inhibition of sirt1 activation by sirtinol reduced protective effects by melatonin against hydrogen peroxide HaCaT keratinocytes were pre-incubated with melatonin (10 μM) and exposed to 800 μM H_2_O_2_ for 18 hr in the presence of sirtinol. The cell viability was measured by crystal violet staining. Viability of the control cells was set at 100%, and viability relative to the control was measured **A. B.** LDH activities were measured by quantifying LDH released in the medium **C.** HaCaT keratinocytes were incubated with melatonin in the presence of sirtinol for 24 hr. Western blot for sirt1 and acetyl-p53 proteins was analyzed from HaCaT keratinocytes. β-actin was used as loading control **D.** Bar graph indicates the mean ± standard error of the mean (SEM) (*n* = 4). * *p* < 0.05, ** *p* < 0.01 significant differences between control and each treatment group and # *p* < 0.01; significantly different when compared with H_2_O_2_ and melatonin-treated group.

**Figure 5:**
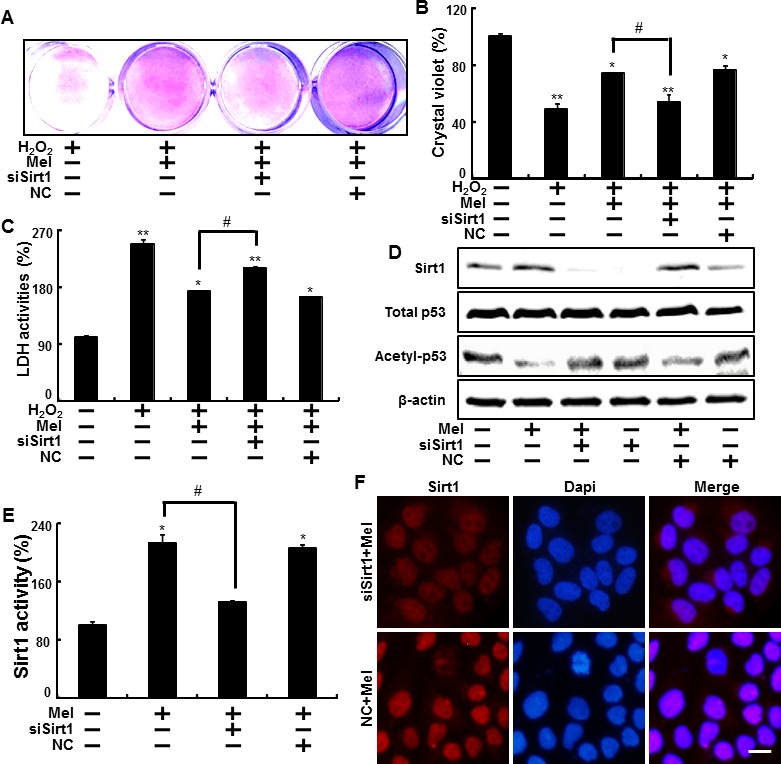
Blocking of sirt1 activity inhibited the melatonin-induced protection against hydrogen peroxide in keratinocyte Sirt1 small interfering RNA (siSirt1) or negative control siRNA (NC siRNA) transfected HaCaT keratinocytes were incubated with 800 μM H_2_O_2_ for 18 hr in the presence of melatonin. The cell viability was measured by crystal violet staining. Viability of the control cells was set at 100%, and viability relative to the control was measured **A.**, **B.** LDH activities were measured by quantifying LDH released in the medium **C.** siSirt1 or NC siRNA (negative control siRNA) transfected HaCaT keratinocytes were incubated with melatonin for 24 hr. Western blot for sirt1 and acetyl-p53 proteins was analyzed from HaCaT keratinocytes. β-actin was used as loading control **D.** Sirt1 deacetylase activities in nuclear were analyzed from HaCaT keratinocytes **E.** The cells were immunostained with sirt1 antibody (red) and observed in the fluorescent view Scale bar: 10 μm **F.** Bar graph indicates the mean ± standard error of the mean (SEM) (*n* = 4). * *p* < 0.05, ** *p* < 0.01 significant differences between control and each treatment group and # *p* < 0.01; significantly different when compared with H_2_O_2_ and melatonin-treated group.

The effect of melatonin on sirt1, which are known as effective factor for H_2_O_2_ in keratinocytes was also examined. In the Figure 4 and 5, to verify the role of sirt1, we further tested the effects of melatonin on sirt1 using sirtinol (sirt1 inhibitor) and sirt1 siRNA.

HaCaT keratinocyte pretreated sirtinol with melatonin displayed a decrease of cell death and sirtinol did not change the cell viability in H_2_O_2_ treated cells (Figure 4A and 4B). And melatonin-induced reduction of LDH activities was inhibited by inactivation of sirt1 with sirtinol in LDH assay (Figure 4C). As shown in Figure 4D, the expression levels of sirt1 were slightly decreased in treated melatonin after pretreating sirtinol and acetyl-p53 also showed differences in melatonin treatment and pretreating sirtinol.

In addition, melatonin effects on H_2_O_2_-induced cell damage were significantly reduced after sirt1 siRNA transfection in H_2_O_2_ treated cells (Figure 5A, 5B). And the effect on decreased LDH secretion from melatonin treatment was blocked by sirt1 siRNA transfection in H_2_O_2_ treated cells (Figure 5C). In protein levels, the silencing of sirt1 with sirt1 siRNA reduced melatonin-induced sirt1 increase, and restored acetyl-p53 to control levels (Figure 5D). Consequently, melatonin up-regulated sirt1 activity, and transfecting sirt1 siRNA blocked increased sirt1 activity caused by melatonin treatment. Sirt1 activity was not changed in the negative control (Figure 5E). Immunocytochemistry for sirt1 showed the cells transfecting negative control exhibited the increased sirt1 levels by treatment of melatonin and sirt1 siRNA-transfected cells treated with melatonin showed attenuated the sirt1 expression (Figure 5F).

Our data suggested that melatonin could effectively enhance sirt1 expression and inhibition of sirt1 with sirtinol and sirt1 siRNA led to reverse the melatonin-induced increased cell viability in H_2_O_2_ treated cells.

**Figure 6:**
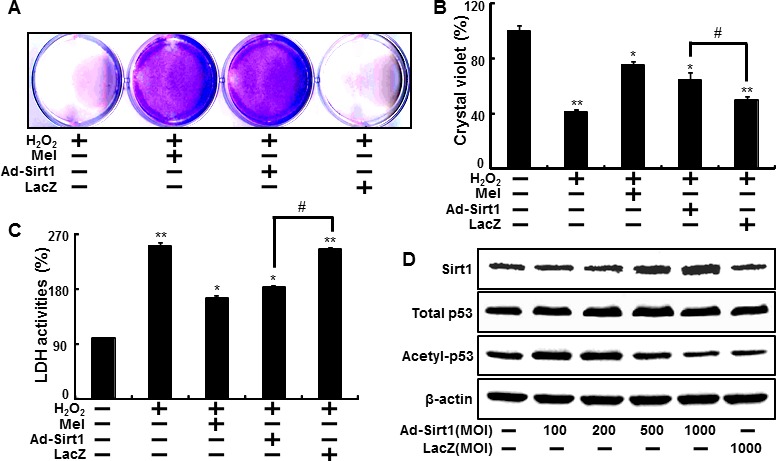
Overexpression of sirt1 increased protective effects against hydrogen peroxide in human keratinocytes HaCaT keratinocyte was transfected by overexpressing adenovirus (Ad-Sirt1) or lacZ-bearing adenovirus (Ad-lacZ) and then exposed to 800 μM H_2_O_2_. Cell viability was measured by the Crystal violet assay. Viability of the control cells was set at 100%, and viability relative to the control was measured **A. B.** LDH activities were measured by quantifying LDH released in the medium **C.** HaCaT keratinocyte was transfected by overexpressing adenovirus (Ad-Sirt1) or lacZ-bearing adenovirus (Ad-lacZ) for 24h. A Western blot for sirt1 and acetyl-p53 proteins was conducted from HaCaT keratinocytes. Beta-actin was used as the loading control **D.** Bar graph indicates the mean ± standard error of the mean (SEM) (*n* = 4). * *p* < 0.05, ** *p* < 0.01 significant differences between control and each treatment group.

**Figure 7:**
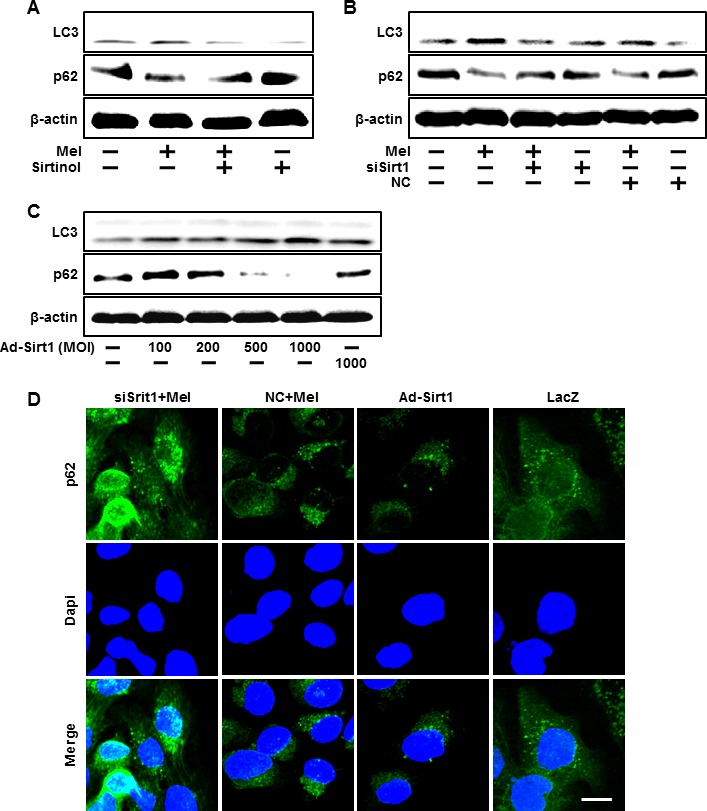
Blocking of sirt1 activity using siRNA and sirtinol inhibited the activation of autophagy flux by melatonin HaCaT keratinocytes were incubated with melatonin in the presence of sirtinol for 24 hr. Total keratinocyte extracts were prepared and analyzed by Western blot for LC3-II and p62 protein levels **A.** Sirt1 small interfering RNA (siSirt1) or negative control siRNA (NC siRNA) transfected HaCaT keratinocytes were incubated with 10 μM of melatonin. Western blot for LC3-II and p62 proteins was analyzed from HaCaT keratinocytes **B.** HaCaT keratinocyte was transfected by overexpressing adenovirus (Ad-Sirt1) or lacZ-bearing adenovirus (Ad-lacZ). Western blot for LC3-II and p62 proteins were analyzed from HaCaT keratinocytes **C.** β-actin was used as loading control. The cells were immunostained with p62 antibody (green) and observed in the fluorescent view Scale bar: 10 μm **D.**

We also investigated whether sirt1 overexpression by adenoviral transfection also have protective effects on hydrogen peroxide. Consistent with melatonin treatment, Ad-sirt1 transfected cells increased the protective effects compared to Ad-LacZ transfected cells (Figure 6A-6C). And at multiplicity of infection (MOI) of 100-1000, Ad-sirt1 gradually increased sirt1 protein expression with protein levels at 100 MOI (Figure 6D). We further investigated whether melatonin-induced sirt1 as an important upstream intermediate of autophagy in HaCaT keratinocytes. Melatonin-induced autophagy was impaired by sirt1 inhibitor, sirtinol (Figure 7A). In addition, sirt1 siRNA significantly reversed the p62 reduction by melatonin treatment (Figure 7B). And also overexpression of sirt1 using adenovirus transfection reduced the p62 protein levels and enhanced LC3-II protein levels (Figure 7C). The results after the p62 antibody immunostaining of HaCaT keratinocytes also showed that melatonin-induced reduction of p62 protein was increased by silencing sirt1, and also overexpression of sirt1 also led to reduction of p62 protein levels (Figure 7D). These results indicate that melatonin-induced autophagy protects the human keratinocytes against hydrogen peroxide through sirt1 activation.

## DISCUSSION

The primary goal of this study was to demonstrate the possibility that melatonin, a major product of the pineal gland [70, 71], may have a preventive effect on human keratinocytes exposed to hydrogen peroxide during the autophagy process via sirt1 activation.

Melatonin, known as *N*-acetyl-5-methoxytryptamine, demonstrates functionality as an important protective factor in various cells [23, 24, 72]. Melatonin by increasing Sirt1 activity and expression could enhance PGC-1 alpha function and mitochondrial biogenesis and function in cadmium-induced hepatotoxicity [73]. And also Sirt1 could have anti-inflammatory effects and cytoprotective effects enhanced by melatonin through the downregulation of PI3K/Akt, MAPKs, and NF-κB signaling [74].

Many studies have focused on the role of melatonin in protecting against UVB-induced apoptosis and oxidative stress as well as seasonal reproduction and immune function [75, 76]. However, it remains unclear whether melatonin prevents hydrogen peroxide-induced cell death and what, if any, protective action it exerts in keratinocytes. Melatonin has also been recognized as a potent scavenger of ROS that exhibits antioxidant effects through a free scavenging cascade and could prevent H_2_O_2_-induced cell damage and local inflammation in the adrenal medulla [10, 77]. However, we were able to verify that, independently of the ROS process, melatonin influences keratinocytes damaged by hydrogen peroxide through autophagy.

The inhibition ofautophagy significantly increases the generation of inflammatory cytokines and p62 expression in primary human keratinocytes [78]. Many reports have addressed the potential roles of autophagy, primarily those related to metabolic stress [79], in human keratinocyte differentiation [77]. Autophagy plays a major role in the degradation of cytoplasmic proteins in cells that are exposed to stressors that facilitate protein aggregation [80]. Protein degradation is induced by autophagy and an autophagosome-lysosome fusion is important in supplying amino acids in situations where there is nutrient starvation [81].

In Figure 3D, we demonstrate that H_2_O_2_ treatment led to autophagy dysfunction. Several previous studies report that hydrogen peroxide enhances the induction of autophagy orautophagic cell death. However, autophagic processes triggered by hydrogen peroxide have not yet been conducted on human keratinocytes, so we cannot be certain of this result. Future studies are needed to analyze autophagy in response to hydrogen peroxide in human keratinocytes. And autophagy is in close connection with Sirt1 pathway. Many studies have shown that Sirt1 promotes autophagy-mediated processes which have positive effects for antiviral immune responses [82], cytotoxicity [83], hepatosteatosis [84] and kidney disease [85]. But relation of Sirt1 and autophagy in keratinocytes did not have been known.

In this study, we also did not confirm the effects of melatonin in primary keratinocytes. Consequently, protective effects of melatonin have yet to be demonstrated in primary human keratinocytes and mouse models. However, many possibilities exist for the use of melatonin as a protective agent against hydrogen peroxide in primary human keratinocytes. In an effort to clarify the potential use of melatonin as a protective tool, we plan to investigate whether melatonin exerts a protective effect against hydrogen peroxide as a result of sirt1/autophagy pathways in primary human keratinocytes and mouse models.

This study recognizes that the cellular and molecular mechanisms of melatonin-induced autophagy play a protective role through sirt1 pathway against skin cell damage as a result of hydrogen peroxide-induced keratinocyte cell death. We suggest that melatonin may act as a therapeutic target in anti-cancer and anti-aging activities.

## MATERIALS AND METHODS

### Cell culture

HaCaT keratinocytes, an immortalized, non-tumorigenic human keratinocyte cell line, were maintained in DMEM supplemented with 10% FBS and 1% antibiotics at standard conditions (37°C, 5% CO_2_, in a humidified incubator). For proliferation studies, HaCaT cells were seeded in 60-mm culture dishes in standard medium or in the presence of different concentrations of melatonin (1, 2.5, 5, 10 μM) and H_2_O_2_ (100, 200, 400, 800 μM). At appropriate intervals, triplicate dishes were trypsinized and cell numbers were determined by counting cell suspension in a Neubauer hemocytometer. Reported values represent the mean ± S.D. of three independent samples at each experimental point.

### Crystal violet assay

Cell viability was evaluated using crystal violet staining. Cells were briefly stained with a staining solution (0.5% crystal violet in 30%ethanoland 3%formaldehyde) for 10 minutes at room temperature (RT) and washed 4 times with water. The stained cells were lysed with 1% sodium dodecyl sulfate (SDS) and absorbance was measured at 550 nm. Cell viability was calculated based on relative dye intensity as compared with the control.

### Lactate dehydrogenase (LDH) assay

Cytotoxicitywas assessed in the supernatants using a LDH cytotoxicity detection kit (Takara Bio; Tokyo, Japan) according to manufacturer's instructions. LDH activity was determined by measuring the absorbance at 490nm using a Spectra Max M2 microplate reader (Molecular Devices; Sunnyvale, CA, USA).

### Western blot analysis

After HaCaT keratinocytes were lysed in buffer (25 mM 4-(2-hydroxyethyl)-1-piperazineethanesulfonic acid, 100 mM NaCl, 1 mM ethylenediaminetetraacetic acid, 5 mM MgCl_2_, 0.1 mM dithiothreitol and a protease inhibitor mixture at pH 7.4), proteins were electrophoretically resolved on 10–15% sodium dodecyl sulfate polyacrylamide gel and transferred to a nitrocellulose membrane. Immunoreactivity was detected through sequential incubations with horseradish peroxidase-conjugated secondary antibodies and enhanced chemiluminescence reagents. Antibodies used for immunoblotting included LC3 (Novus Biologicals, Littleton, CO, USA), p62 (Millipore, Bedford, MA, USA), autophagy related 5 (ATG5) (Abcam, Cambridge, MA, USA), SIRT1 (Santa Cruz Biotechnology), acetyl-p53 (Santa Cruz Biotechnology), and β-actin (Sigma Aldrich, St. Louis, MO, USA). Images were examined using a Fusion FX7 imaging system (Vilber Lourmat, Torcy Z.I. Sud, France).

### RNA interference

HaCaT keratinocytes were transfected with ATG5 small interfering RNA (siRNA; oligoID HSS114104; Invitrogen, Carlsbad, CA, USA) and SIRT1 siRNA (oligoID VHS50608; Invitrogen) using Lipofectamine 2000 according to manufacturer's instructions. After a 48-hour culture period, knockdown efficiency was typically measured at the protein level using immunoblot analysis. Nonspecific siRNA (oligoID 12935–300; Invitrogen) was used as a negative control.

### Construction of recombinant adenoviruses

The Sirt1 over-expressing adenovirus (Ad-Sirt1) was provided by Professor Byung-Hyun Park of Chonbuk National University (Jeonju, Jeonbuk, South Korea). The lacZ-bearing adenovirus (Ad-lacZ) was used as a control. Recombinant adenoviruses were amplified in human embryonic kidney (HEK)-293 cells and purified using the Vivapure AdenoPACK kit (Sartorius AG, Göttingen, Germany) according to manufacturer's instructions.

### Immunocytochemistry

HaCaT keratinocytes were cultured on glass coverslips positioned on a 24-well plate. Cells were washed withphosphatebuffered saline (PBS) and fixed with cold acetone for 90 seconds. Following this, cells were washed with PBS, blocked with 5% FBS in Tris-buffered saline with Tween, and incubated with anti-p62 monoclonal antibodies (2 μg/mL) or anti-sirst1 polyclonal antibodies (5 μg/mL) (for 48 hours at RT (20°C). Unbound antibody was removed with a PBS wash and cells were incubated with Alexa Fluor 488anti-mouse IgGantibody (2 μg/mL) or Alexa Fluor 546 anti-rabbit IgG antibody (2 μg/mL) for 2 hours at RT. Finally, cells were mounted with DakoCytomation fluorescent medium and visualized using afluorescence microscope.

### Sirt1 deacetylase activity assay

To measure cellular Sirt1 deacetylase activity, nuclear proteins were extracted from HaCaT keratinocytes using a Nuclear/Cytosol Fractionation kit (BioVision, Milpitas, CA, USA). Sirt1 deacetylase activity was quantified following the protocols of the Sirt1 Fluorometric Assay kit (Sigma-Aldrich). Fluorescence intensities were measured with a microplate fluorometer (excitation wavelength = 360 nm, emission wavelength = 450 nm). The fluorescence intensities of Sirt1 deacetylase activity were normalized to the protein levels measured in the cell samples.

### Statistical evaluation

All data are expressed as the mean±standard deviation and were compared using Student'st-test, analysis of variance and Duncan's test using SAS statistical software version 9.1 (Version 9.1, SAS Institute, Cary, NC, USA). Results were considered significant at*p<0.05, **p<0.001 and # *p* < 0.01 as appropriate.
